# Bacteriology in Medicine and Surgery

**Published:** 1900-06

**Authors:** 


Bacteriology in Medicine and Surgery.—A practical manual for physicians, health officers and students. By Wm. Hallack Park, M. D., Associate Professor of Bacteriology and Hygiene, University and Bellevue Hospital Medical College, and Assistant Director of the Research Bacteriological Laboratories of the Department of Health, City of New York; assisted by A. R. Guerard, M. D., Assistant Bacteriologist, Department of Health, City of New York. Illustrated with 87 engravings and two colored plates. Pages, 693. Price, $3.00. Lea Brothers & Co., New York and Philadelphia. 1900.

The book is especially suited to the general practitioner and the health officer. The facts in bacteriology have been grouped in such way as to make the subject easy of comprehension by the beginner, and of great importance as a reference by those who are better advanced. The essential technique for all practical examinations in the small laboratory of the physician, as well as in the more extensive work of the investigator, could not be more clearly laid down. Great emphasis is placed upon the chemical changes produced by bacteria, infection, immunity, the nature and use of protective serums, and the diagnostic value of bacteriological cultures. The practitioner must now-a-days be able to take advantage of this information. This book should have a large sale among the general practitioners and health officers throughout the country. T. J. B.

